# Nitric Oxide and Lutein: Function, Performance, and Protection of Neural Tissue

**DOI:** 10.3390/foods4040678

**Published:** 2015-11-11

**Authors:** James M. Stringham, Nicole T. Stringham

**Affiliations:** Nutritional Neuroscience Laboratory, Department of Psychology, University of Georgia, Athens, GA 30602, USA; E-Mail: ntwood@uga.edu

**Keywords:** lutein, nitric oxide, oxidative stress, inflammation

## Abstract

The soluble gas neurotransmitter nitric oxide (NO) serves many important metabolic and neuroregulatory functions in the retina and brain. Although it is necessary for normal neural function, NO can play a significant role in neurotoxicity. This is often seen in disease states that involve oxidative stress and inflammation of neural tissues, such as age-related macular degeneration and Alzheimer’s disease. The dietary xanthophyll carotenoid lutein (L) is a potent antioxidant and anti-inflammatory agent that, if consumed in sufficient amounts, is deposited in neural tissues that require substantial metabolic demand. Some of these specific tissues, such as the central retina and frontal lobes of the brain, are impacted by age-related diseases such as those noted above. The conspicuous correspondence between metabolic demand, NO, and L is suggestive of a homeostatic relationship that serves to facilitate normal function, enhance performance, and protect vulnerable neural tissues. The purpose of this paper is to review the literature on these points.

## 1. Nitric Oxide in Normal Retinal Function

**Figure 1:**
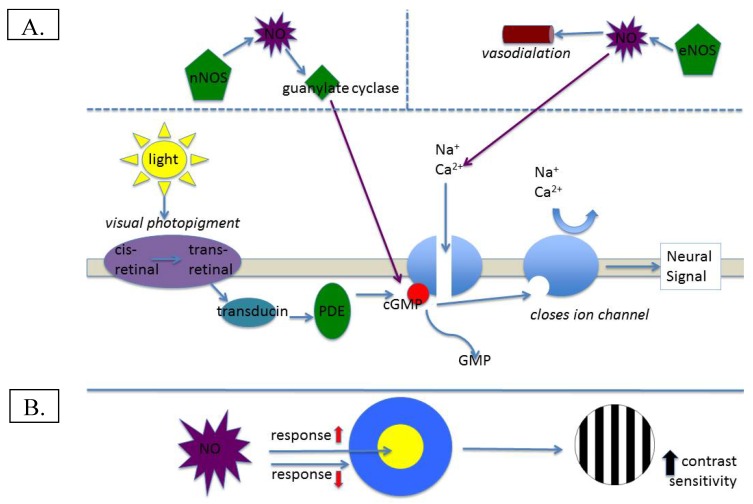
(**A**) Illustration of normal involvement of NO in visual transduction; (**B**) Potential effect of NO on contrast sensitivity.

The retina maintains the highest metabolic rate of any tissue found in the body [1]. Illustrative of this point, the oxygen consumption of the mammalian retina (per gram of tissue) is three times greater than that of the cerebral cortex, and six times that of cardiac muscle [2]. The physiology of the retina is not only rapid, but requires complex processes to achieve its function of transducing photons of absorbed light into trains of neural impulses that ultimately produce vision. Classically referred to as the “visual cycle”, this process involves at least 10 distinct molecular steps, each of which involves complex interactions between numerous molecules. Nitric oxide (NO), first discovered in the retina roughly 25 years ago [3], has been shown to serve many important functions for retinal physiology. NO is generated by the interaction of the amino acid arginine and three precursor synthase molecules [4]: endothelial, neuronal, and inducible (eNOS, nNOS, and iNOS respectively), with eNOS and nNOS being calcium-dependent, whereas iNOS is calcium-independent. Each of the synthases is related to specific function for NO, or, in the case of iNOS, triggered by inflammation or pathological states [5]. NO generated by eNOS serves the function of vasodilation and nNOS is related to calcium-based demand for NO in the visual cycle [6] (see below). Given its vasodilatory effects, one important way that NO affects visual physiology is by regulating retinal blood flow. The well-characterized ability of NO to act as a rapid vascular endothelial relaxant (e.g., [7]) provides the retina with adequate nourishment for its relatively prodigious metabolic appetite. Further downstream, NO acts to regulate and enhance visual physiology. In terms of visual adaptation, NO (via synthesis by nNOS) has been shown to stimulate guanylate cyclase production, which leads to formation of cyclic guanosine monophosphate (cGMP). In the vertebrate retina, cGMP mediates photoreceptor signal transduction and modulates ion channel and gap junction conductivity [8]; specifically, cGMP serves to hold sodium channels open ([6]; see Figure 1A). This important function ensures that a photoreceptor is capable of stimulation by incoming photons, and modulates calcium channel currents, which could fine-tune or even enhance visual sensitivity [9]. Indeed, this function is widespread in the retina, as NO has been shown to modulate light responses in all retinal neuron classes via specific ion conductance activation [10]. Strong, unequivocal supporting evidence for its role in normal visual function was found by Ostwald *et al.* [11], who showed that dark-adapted electroretinogram responses to single flashes of light were significantly reduced in cats who had been administered a nitric oxide synthase (NOS) inhibitor. Moreover, response amplitudes to dim and bright flashes of light have been shown to increase in the presence of NO [12]. Lastly, in horizontal cells, responses to stimulation of the surround of the receptive field have been shown to be reduced, whereas responses to stimulation of the center of the receptive field are increased in the presence of NO [12]. This situation would manifest visually as increased contrast sensitivity, and could provide a NO-based explanation for depressed contrast sensitivity in those with visual disease (see below). Certainly all of the available evidence indicates that NO is necessary for normal visual function, and may actually enhance some aspects of visual function.

## 2. Nitric Oxide in Retinal Disease

Although NO is clearly used in the retina to maintain normal visual function, it has also been found to be related to ocular disease states involving ischemia (such as diabetic retinopathy [13]), oxidative stress (such as age-related macular degeneration (AMD); e.g., [14]), and inflammation (including diabetic retinopathy, AMD, and retinitis pigmentosa (e.g., [15]). The nature of the relationship between NO and the aforementioned diseases is that increased levels of NO are typically associated with markedly worse disease states. Consequently, there appears to exist a paradox of sorts, whereby NO is shown to be necessary for normal (and perhaps enhanced) visual performance, but is also associated with many forms of ocular disease. The derivation of why and how NO is associated with (and can lead to disease states) in the retina has the potential to elucidate not only mechanisms of disease, but also plausible ways to retard disease progression, including dietary modification.

**Figure 2:**
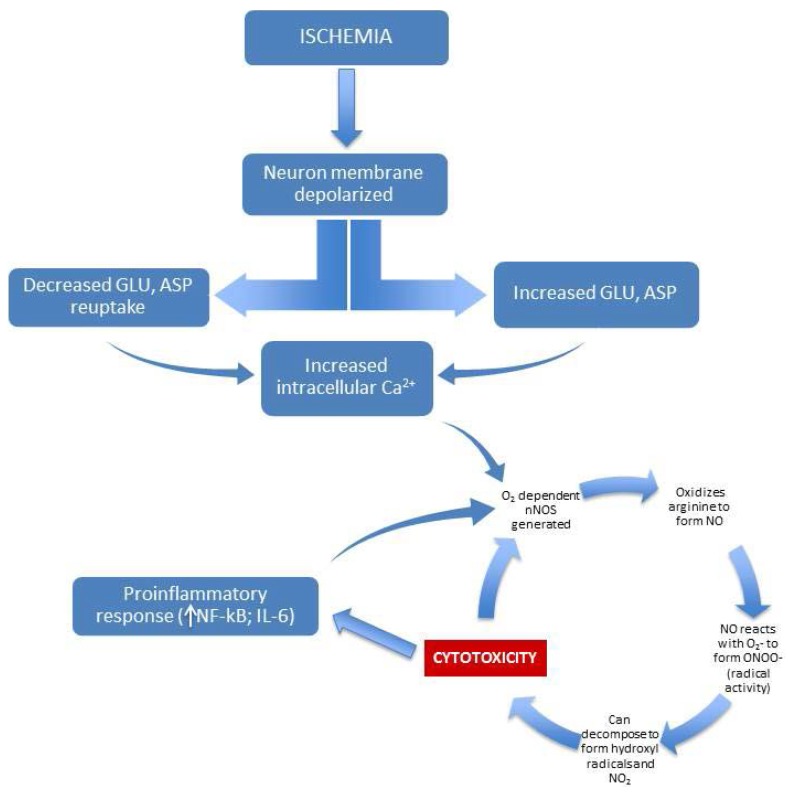
Illustration of NO-based excitotoxic/inflammatory cycle mediated by ischemia.

One well-characterized mechanism of pathological activation of NO involves excitotoxicity associated with ischemia and consequent reperfusion injury [16]. Retinal ischemia, for example, can occur as a result of virtually any disease that affects retinal blood supply, such as diabetes mellitus or AMD [17]. The mechanism by which ischemia leads to toxic effects in neural tissue involves the dysregulation of glutamate and aspartate metabolism [18]. Ischemia produces dramatic increases in these excitatory neurotransmitters, coupled with an apparently reduced capacity for reuptake [19]. The excess glutamate and aspartate leads to increases in intracellular calcium and free radical oxygen activity, which has been convincingly shown to lead to cell death by destruction of DNA, proteins, and the cell membrane [20]. The end result is neurotoxicity, which can (for the case of the retina) result in blindness. The connection between ischemic injury and NO is significant. Once released, glutamate and aspartate bind to and stimulate NMDA receptors, and this leads to the aforementioned increases in intracellular calcium levels—importantly (because nNOS is calcium-dependent), this causes nNOS to produce NO [21,22,23]. The entire cycle points to the action of free-radical oxygen as a major factor in neurotoxicity. If oxidative stress does not lead directly to neurotoxicity, it can permanently modify cellular function. For example, oxidative stress brought on by strongly reactive oxygen species (e.g., free radicals and peroxides) can lead to long-term damage to cell DNA, which can manifest as cancer [24]. Additionally, production of iNOS is stimulated by the inflammatory state of the neural tissue, and this leads to further increases in NO [5]. In fact, evidence indicates that the body uses iNOS expression as a cytotoxic defense mechanism [25], where the free-radical oxygen species produced by NO can disrupt and/or kill invading microorganisms [26]. Unfortunately, the increase in inflammatory-based NO can be a potent mediator of neurotoxicity, especially in situations of chronic inflammation ([27,28]—see Figure 2). Strong evidence for this was found by Dawson *et al.* [29], where blockade of NO precursors effectively prevented neurotoxicity. Blockade of cGMP precursors did not reduce neurotoxicity, and so the substantial increase in cGMP typically seen with increases in NO does not appear to be a factor in cell death. With regard to reperfusion injury, again oxidative stress appears to be the driving factor in neurotoxicity. Restoration of circulation after ischemia can lead to many potential oxidative-stress-based mediators (hydrogen peroxide, superoxide, and hydroxyl radicals) of neuronal stress and death [30].

Although NO itself is a radical, its reactivity is low compared to the potentially damaging oxidative products that it generates. N_2_O_3_ and peroxynitrite (ONOO−) are produced by the reaction of NO with O_2_ or superoxide (O_2_−), respectively [31], and have been shown to exert neurotoxic effects [32]. Although peroxynitrite is technically a non-radical, it is a potent oxidant that can react with biomolecules and produce nitration reactions, which can lead to cell death [33]. In a dysregulated state, such as that found in neural disease, excessive production of NO leads to excessive oxidative stress. Consequently, one can deduce that the apparent retinal NO paradox is not a paradox at all, but actually one system working under vastly different conditions: A state of relative homeostatic balance in one case, and a state of dysregulation in the other.

## 3. Lutein and Nitric Oxide: A Homeostatic Symbiosis

The suggested paradox above is not meant to imply that the situation in the retina is either functioning perfectly, or compromised by disease. It is most certainly the case that “normal” function of the retina swings somewhere between these two poles. Because of the wildly fluctuating nature of the physiology of vision, where responses to light levels span a stunning 14 orders of magnitude [34], the second-to-second production and demand for NO most certainly varies significantly. It is apparent in cases where excess NO is produced (such as ischemia or disease states (see above)) that the role of NO in neurotoxicity is significant. Given the varying demand of retinal metabolism, vision under even normal circumstances could plausibly produce cellular damage via NO-based oxidative products; this damage could accumulate and would not manifest as decreased visual performance or clinical retinal damage until advanced age. It would be advantageous therefore to have in place a regulatory system that could quench NO radicals locally, and limit acute and cumulative damage. As a component of the diet-derived macular pigment (MP) in the retina, the excellent antioxidant/anti-inflammatory lutein (L) could plausibly serve this purpose. Although L is the primary carotenoid component of MP [35], zeaxanthin and mesozeaxanthin (a stereoisomer of zeaxanthin, converted from L in the retina [36]) are also significant components of MP [35] and presumably confer the same benefits to neural tissue. MP is most dense in the metabolically intense central retina (fovea), where its powerful antioxidant [37] and high-energy short-wave light filtration [38] properties appear to protect the macula from acute damage [39], protect against cumulative damage resulting in age-related macular disease such as AMD [40], and maintain visual sensitivity over a lifetime [41]. MP is strictly derived via diet, and so a person’s level of MP is dependent upon their consumption of foods that contain these carotenoids. Although found in a variety of colored fruits and vegetables, L is most richly concentrated (~5–10 mg/serving) in leafy-green vegetables such as kale or spinach [42]. L belongs to the subtype of carotenoids known as xanthophylls, designated as such by their molecular structure—xanthophylls exhibit a six-carbon ring that contains oxygen at each end of their carbon-conjugated double bond chains [43]. This structure allows xanthophylls to more easily interact with singlet oxygen species than non-xanthophyll carotenoids, and enables exceptional antioxidant capability: By virtue of their carbon-conjugated double bonds, xanthophylls can “quench” the energy of damaging singlet oxygen and other free radical species through a process called triplet excitation transfer [44]. In sum, this process involves energy transfer reactions in which the energy of singlet oxygen and other free radicals is transferred to carotenoid molecules in the ground state, which results in the formation of triplet-state carotenoid molecules. The energy acquired by the carotenoids is then lost as heat, and the ground state carotenoid is regenerated to undergo another cycle of protection [37]. This property of regeneration is not true of all carotenoids, and makes xanthophylls especially potent antioxidants, capable of long-term accumulation and protection in target tissues such as the retina. Indeed, L has been shown to effectively scavenge superoxide radicals, hydroxyl radicals, and inhibit in-vitro lipid peroxidation [45]. Importantly, a significant reduction in lipid peroxidation by L has been demonstrated *in vivo* for newborn infants, who are at substantial risk of oxidative stress, given the intense metabolic nature of development and low stores of diet-based antioxidants [46,47]. Moreover, L has been found to have meaningful anti-inflammatory action [48,49], which could serve to modulate the many negative effects of inflammation associated not only with injury and disease, but also that brought on by psychological stress [50].

**Figure 3:**
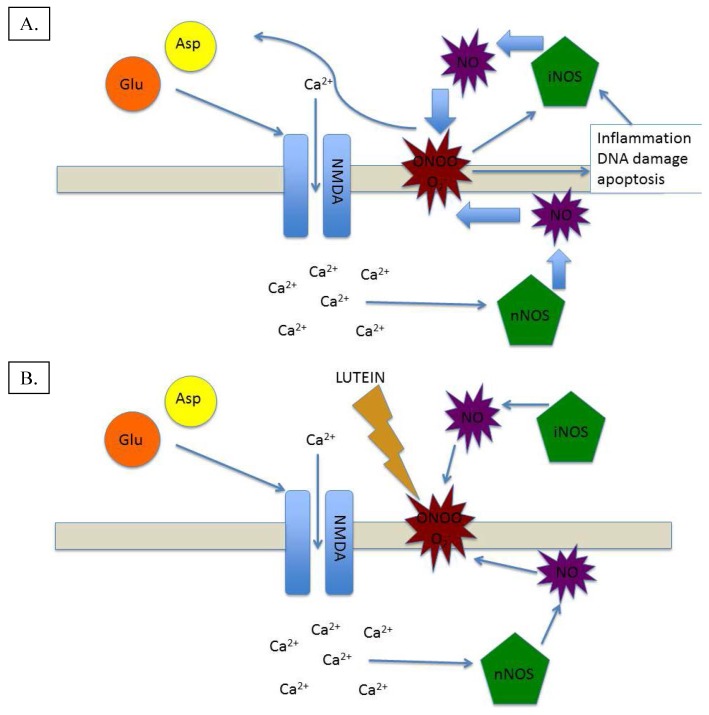
(**A**) Excitotoxic/Inflammatory damage mediated by NO-based generation of oxidative and inflammatory species; (**B**) Excitotoxic/Inflammatory cycle broken by lutein’s antioxidant and anti-inflammatory action.

Beyond the retina, L has also been found to accumulate in the brain [51,52], where it is the dominant carotenoid in both infant [52] and geriatric [51] brain tissues. In a manner apparently similar to the retina, L crosses the blood-brain barrier and accumulates in the brain regions that maintain relatively high metabolism (e.g., frontal and occipital lobes, and hippocampus), and are therefore at higher risk for oxidative stress and inflammation [53]. Importantly, the concentration, or optical density, of MP (MPOD) has been shown to be significantly correlated with brain levels of L and Z [53], which suggests a similar mechanism of uptake, and supports the idea that there is preferential deposition of these powerful antioxidants/anti-inflammatories in neural tissues that maintain high metabolism, concomitant oxygen tension, and potential for inflammation (see Figure 3A,B). The protection of neural tissue afforded by the lifelong accumulation of L in the brain appears to confer a substantial benefit to cognition: MPOD (related to brain levels of L, as noted above) has been shown to be significantly related cognitive performance, especially in aged individuals [54,55,56].

There is much functional, anatomical, and neuroprotective-based evidence to support a form of symbiosis between NO and L. The nature of the relationship between NO and L in retinal and brain tissue most certainly involves the ability of L to quench oxidative products generated by NO. Indeed, it has been shown that L is a very effective antioxidant against N_2_O_3_ and peroxynitrite [45]. Due to its local concentration in areas of the eye and brain that can reach very high metabolic demand and exhibit very high rates of change in metabolic rate, L is optimally positioned to exert its antioxidant effect. Additionally, there is much evidence to suggest that L significantly reduces inflammation associated with lipopolysaccharide (LPS)-induced NO production [57,58]. Rafi and Shafaie [57] showed that L reduced inflammation-based NO production by 50%, and significantly inhibited iNOS gene and protein expression. Speaking more directly to L’s mechanism of action in the inflammatory pathway, Wu *et al.* [58] showed convincingly that the inflammation in LPS-stimulated microglial cells was significantly reduced by L via inactivation of nuclear factor kappa B (NF-κB). Because microglia effectively monitor the immune status of the central nervous system and are capable of inducing inflammation in response to oxidative stress or degenerative diseases [59], the finding that L can suppress this response is significant in terms of neural function and health.

It appears therefore that L can act as a significant modulator of NO-based oxidative products, and can also break the chain of the inflammatory pathway, via gene- and protein-expression changes. For neural health and performance, these functions are significant. In terms of normal visual function and enhancement, there are conspicuous functional and location-based relationships between NO and L that lend further to their apparent symbiosis. Earlier in the manuscript, it was noted that NO modulates ion channel and gap junction conductivity [8]. Coincidentally, L has been shown to enhance gap junction communication [60], via influencing the expression of certain genes or acting as inhibitors of regulatory enzymes. It could be that the two molecules form a system, whereby (due presumably to a reduction in oxidative-stress-based visual cycle inhibition) the visual cycle is allowed to operate at high efficiency. This idea was postulated recently by Stringham *et al.* [61], whose subjects exhibited significantly faster dark adaptation as a function of MPOD. A significant enhancement of physiological function by L has also been demonstrated for speed of visual processing [62,63], where subjects with higher MPOD were shown to have significantly higher critical flicker fusion (CFF) thresholds. Both dark adaptation and CFF rely heavily upon specific aspects of the visual cycle, such as ion conductance activation via cGMP [10], and enzymatic processes that facilitate photopigment regeneration. As noted earlier, both of these physiological parameters are greatly impacted by NO. Given their respective locations, functions, and biochemical properties, this correspondence is suggestive of a functionally mutual dependence between NO and L.

## 4. Conclusions and Future Directions

The evidence for the respective roles of NO and L in visual function and performance is very strong. There is also much evidence to suggest a functional relationship between these two molecules, in which it appears that appropriate levels of each can lead to enhanced visual function. The question of “appropriate” levels for NO and/or L can be addressed indirectly by examining the case of pathology: NO levels and their associated oxidative and inflammatory products tend to be very high in disease states, whereas concentrations of L in the same affected tissues tends to be low. Because L is entirely of dietary origin, it could be the case that low dietary intake of foods that contain L could compromise the homeostatic symbiosis between NO and L described above. Indeed, the National Health and Nutrition Examination Survey (NHANES 2003–2004) [64] indicates that, on average, American adults consume only about 1.5 mg of L/day. As noted above, a typical serving of spinach or kale typically yields at least 5–10 mg of L. The average American diet appears, therefore, to be significantly deficient in L. Moreover, the NHANES found that children between the ages of 1 and 13 years consume 0.3 mg L/day on average. Given the metabolic demands of development, this low intake is worrisome. Also, considering the ability of L to accumulate in tissues such as the retina and brain, low intake early in life may predispose a person to develop age- and oxidative stress-related diseases, such as AMD or perhaps even Alzheimer’s disease [65].

In terms of the function and interaction of NO and L, the retina is the best-characterized neural tissue to date. The brain, however, holds much promise for NO and L research. Although the retina is different from brain tissue in some important ways, it is actually an extension of the brain, and operates using very much the same machinery and similar functional principles [66]. Like in the retina, NO has well-established roles in brain tissue, both in terms of function and neurodegenerative disease [67,68]. The data on L and its role in the brain (described earlier) are encouraging, but there is much we do not know. Given, however, the similarity between the retina and brain, it is tempting to extend the functional and neuroprotective benefits found for L in the retina to the brain. Indeed, the recent cognitive and dementia/Alzheimer’s disease—based data for L are indicative of similar performance and neuroprotective effects as those found in the retina. Importantly, all of the data, without exception, point to the plausibility of L’s complementary role with NO in normal and enhanced neural function, and protection against oxidative- and inflammatory-stress related disease.
